# Trends in Uninsured Clients Visiting Health Centers Funded by the Title X Family Planning Program — Massachusetts, 2005–2012

**Published:** 2014-01-24

**Authors:** Marion Carter, Kathleen Desilets, Lorrie Gavin, Sue Moskosky, Jill Clark

**Affiliations:** 1 Division of STD Prevention, National Center for HIV/AIDS, Viral Hepatitis, STD, and TB Prevention, CDC; 2 Office of Population Affairs, US Department of Health and Human Services; 3 Division of Reproductive Health, National Center for Chronic Disease Prevention and Health Promotion, CDC; 4 Family Planning Program, Massachusetts Department of Public Health

In 2006, Massachusetts passed legislation that broadened access to health insurance for its residents. The percentage of the state population that had health insurance (obtained through either private insurance or publicly funded programs) subsequently increased, reaching 97% in 2011, leaving only 3% uninsured, compared with approximately 9%–20% uninsured among nonelderly residents in 2006 (1). Given such high rates of insurance coverage, questions arise about the need for categorical public health programs designed to serve clients without health insurance. This report describes trends in the percentage of uninsured clients seen at community-based organizations in Massachusetts that received federal funding for one such program, the Title X family planning program. Title X program data from 2005–2012 indicate that client volume remained high throughout the period, and that the percentage of clients who were uninsured declined, from 59% in 2005 to 36% in 2012. Across years, young adults aged 20–29 years and persons whose incomes were 101%–250% of the federal poverty level were more likely to be uninsured than were persons in other age and income groups. After health-care reform, publicly funded family planning services in Massachusetts saw continued demand from uninsured and insured clients. Family planning services in other states implementing health-care reform might have a similar experience, and public health agencies are encouraged to track such trends to monitor the demand for such services and inform budget planning and resource allocation.

Annual program monitoring data for 2005–2012 were obtained from organizations funded by Title X in Massachusetts. The Title X Family Planning Annual Report (FPAR) data system collects information annually from all entities that receive grants from the Title X appropriation. FPAR includes data on the number and percentage of all family planning clients who did and did not have health insurance that covered a broad set of primary care benefits at the time of their last visit. In this definition, coverage for only limited primary care services, such as that obtained through some Medicaid family planning expansion programs, would not be considered insurance coverage. Also, for this report, the term “health insurance” is used to include coverage obtained through either private insurance companies or publicly funded programs such as Medicaid.

In Massachusetts, health centers obtained health insurance information directly from clients and entered it into a centralized regional data system, either directly or by exporting from electronic systems. The Title X Region One office received and processed that information. Data for clients whose insurance status was unknown (≤3% of total clients across all years) are not presented. FPAR also includes data on self-reported income and age of clients. The regional system stores records of each clinic visit, allowing cross-tabulation of these variables.

Of the six health-care organizations in Massachusetts that directly received grants under the Title X program at any time during 2005–2012, five were funded in any one of those years, and four were funded continuously (Figure 1). In one region of the state, the grantee changed in 2010 from one organization to another (B and F). Each organization oversaw a network of health centers, ranging from five (grantee F) to 51 clinical locations (grantee E). The health centers offered family planning and other preventive services, such as cervical and breast cancer screening, screening for hypertension, and sexually transmitted disease and human immunodeficiency virus testing.

Of the five grantees in 2012, three (A, C, and D) were relatively small, not-for-profit agencies that focused on reproductive health either exclusively or as part of a mix of health and social services. One (F) was a nonprofit reproductive health organization affiliated with a national network, which became a Title X grantee in 2010. The last was a social services agency that offered family planning services through a network of community health centers and other health-care providers in the greater Boston area (E). Together they served low-income clients across the state.

During 2012, the health-care organizations saw 66,227 family planning clients, which was 90% of their 2005 client volume. Grantees varied in client volume, with the number of unduplicated clients served ranging from 9,037 to 29,921 in 2012. Of the four organizations that were continuously funded during this period, one saw a 1% increase in clients, whereas the others experienced decreases of 6%, 7%, and 30%.

From 2005 to 2012, the percentage of clients served by Title X–funded organizations in Massachusetts who were uninsured declined from 59% to 36% (Figure 1). Each of the grantees reported decreases in the percentage of family planning clients without health insurance in this period. In 2005, the percentage of clients who reported not having health insurance ranged from 77% (A) to 46% (E). By 2012, those without health insurance ranged from 52% (A) to 24% (E).

All age groups experienced similar decreases in the percentage of persons who reported they had no health insurance (Figure 2). In each year examined, adults aged 20–29 years comprised 43%–46% of all clients seen by these health centers. They also were the most likely to be uninsured throughout the period. However, they showed the greatest decrease in the percentage lacking insurance (65% uninsured in 2005, 39% in 2012), followed by teens (56% uninsured in 2005; 31% in 2012).

The percentage without insurance declined in all income groups (Figure 3). Throughout the period, however, clients with incomes of 101%–138% and 139%–250% of the federal poverty level (FPL) had the highest percentages without insurance (46% and 43%, respectively, in 2012).

## Editorial Note

The results of this study indicate that in the 6 years following health-care reform in Massachusetts, publicly funded providers continued to be used as providers of choice for many clients with health-care coverage and remained as a “safety net” for uninsured persons in need of family planning services. For these family planning providers, implementation of state health-care reform coincided with significant decreases in the percent of their clients without insurance, although that proportion remained significantly higher than in the general population. The proportion of uninsured clients at safety-net family planning providers dropped significantly within 2 years of reform. However, it then began to level off, remaining over 23% for each health-care organization 6 years after reform. Community health centers and the substance abuse treatment sector in Massachusetts experienced similar shifts (2,3). Conservative estimates from other federal programs such as the Breast and Cervical Cancer Screening Program indicate that large numbers of women will continue to qualify for those subsidized services after insurance expansion (4).

The continued provision of safety-net family planning services is important not just for the individual clients accessing services at these organizations but for broader health equity goals as well. Adults aged 20–29 years experience the most unintended pregnancies of any age group in the United States (5), and these clients constitute a large proportion of clients seen by these health centers. Yet insurance coverage among these young adults lagged behind that of other age groups. Within this client population, near-poor clients (clients whose incomes were above 100% FPL but below 250% FPL) were most likely to be uninsured. The Title X program was designed to serve poor and near-poor clients, the same population typically served by means-tested Medicaid family planning expansion programs (6). This analysis indicates that these income groups might still need access to supportive and safety-net services, even after health-care reform.

Research explaining these trends is sparse and raises questions about why so many clients at Title X–funded health centers lacked health insurance years after reform. Young adults are known to be among those groups with the lowest insurance coverage in Massachusetts and might opt to remain without insurance (7). Newly covered persons might experience difficulty maintaining enrollment in health insurance plans because of strict eligibility rules and changing life circumstances. Those persons might seek services at safety-net providers during periods when they are without health-care coverage (8). Others might not use their insurance as intended because they do not understand its family planning coverage, they seek a service that is not covered by their insurance, or they cannot afford the copayments (3,8).

There is also a role for safety-net providers to serve those who are insured, many of whom might prefer those providers (9). Insured adolescents and young adults might seek subsidized family planning services because they want to keep their visits out of health insurance records and confidential from parents (9,10). Other newly insured clients might not be able to access new primary care providers offered in their insurance networks in a timely way, and thus will continue to seek services from safety-net providers (2,10). As the proportion of clients with insurance who use safety-net providers increases, organizations such as these will have to consider how to rapidly forge new relationships with private insurers and expand their third-party billing capacity.

The findings in this report are subject to at least three limitations. First, FPAR data on insurance, age, and income are self-reported and prone to response bias. Second, the analysis examined trends associated with, but not necessarily caused by, the timing of health-care reform in Massachusetts. Finally, the health-care context in Massachusetts is exceptional in many ways and limits the generalizability of these findings to other states.

Anticipating the effects of health reform in other contexts is difficult. In other states, the percentage of uninsured family planning clients seen at Title X–funded organizations might be significantly higher after a similar period of reform. That might occur in states with 1) higher baseline percentages of uninsured persons, 2) more undocumented immigrants, 3) less expansive public insurance options, 4) less state support for family planning, or 5) a less developed system of community health centers. In other states, the percentage of uninsured clients might be lower, depending on the status of these and other factors. Nevertheless, the experience of Massachusetts highlights the benefit of carefully monitoring the use of publicly funded family planning services in the years following implementation of health reform, and to continue to provide those services, as needed. Additional research and evaluation is critical to better understand the factors affecting trends among uninsured clients that Title X–funded providers, and other safety-net providers, will continue to see as health insurance coverage expands across the United States.

What is known about this topic?

In Massachusetts, health-care reform enacted in 2006 made health-care insurance coverage nearly universal. How health-care service use will change when insurance coverage expands is unclear, particularly for health-care providers who serve as a safety-net for uninsured clients.

What is added by this report?

Data provided by health-care providers funded through the federal Title X family planning program in Massachusetts demonstrate that the percentage of clients who were uninsured decreased significantly during the 6 years since enactment, but the demand for safety-net family planning providers has continued.

What are the implications for public health practice?

Ongoing monitoring of the use of publicly funded family planning services is needed after expansion of enrollment in health insurance.

## Figures and Tables

**FIGURE 1:**
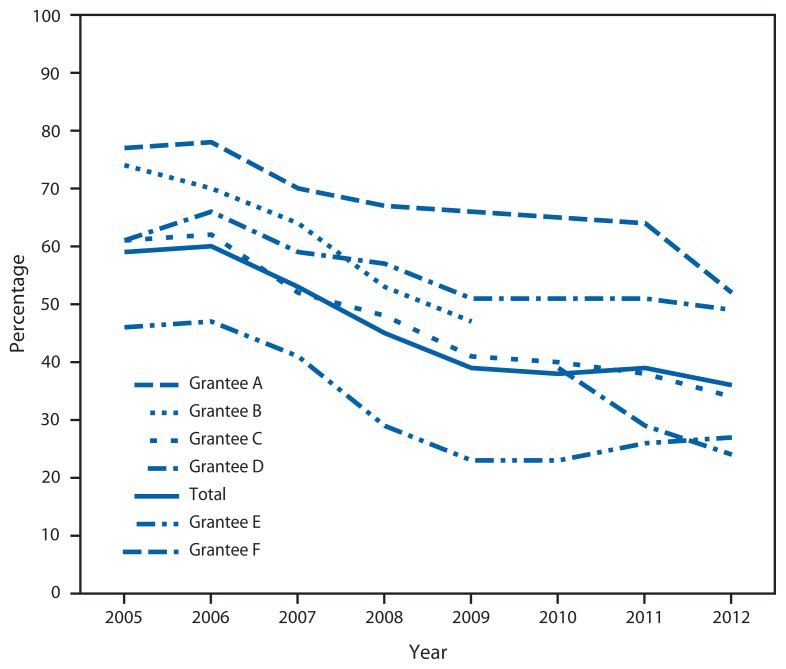
Percentage of family planning clients without health insurance among Title X-funded health centers, by grantee — U.S. Department of Health and Human Services Region One Family Planning Annual Report, Massachusetts, 2005–2012

**FIGURE 2:**
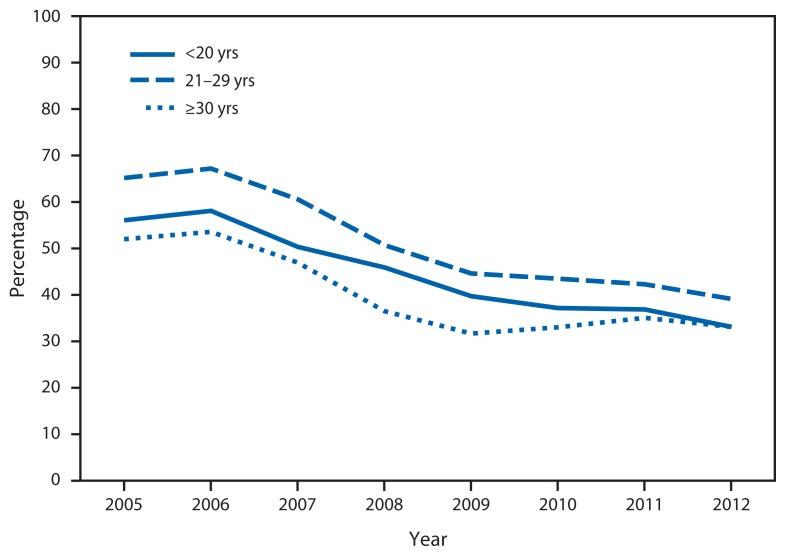
Percentage of Title X clients without health insurance, by age group — U.S. Department of Health and Human Services Region One Family Planning Annual Report, Massachusetts, 2005–2012

**FIGURE 3:**
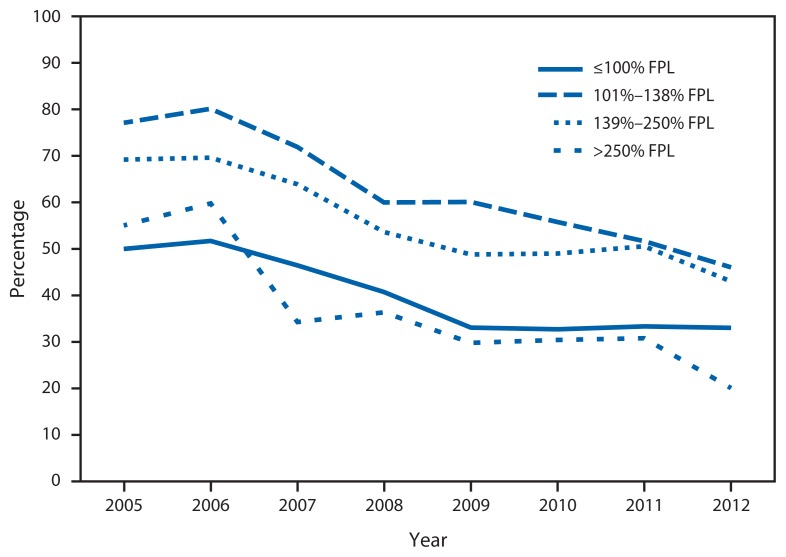
Percentage of Title X clients without health insurance, by federal poverty level (FPL) — U.S. Department of Health and Human Services Region One Family Planning Annual Report, Massachusetts, 2005–2012
